# Alkylating HIV-1 Nef - a potential way of HIV intervention

**DOI:** 10.1186/1742-6405-7-26

**Published:** 2010-07-26

**Authors:** Yong-Jiu Jin, Xiaoping Zhang, Catherine Yi Cai, Steven J Burakoff

**Affiliations:** 1 Department of Oncological Sciences, Mount Sinai School of Medicine, New York, NY 10029, USA; 2 Department of Pharmaceutics, Rutgers University, School of Pharmacy, Piscataway, NJ 08854, USA; 3 Cancer Institute, Mount Sinai School of Medicine, New York, NY 10029, USA

## Abstract

**Background:**

Nef is a 27 KDa HIV-1 accessory protein. It downregulates CD4 from infected cell surface, a mechanism critical for efficient viral replication and pathogenicity. Agents that antagonize the Nef-mediated CD4 downregulation may offer a new class of drug to combat HIV infection and disease. TPCK (N-α-p-tosyl-L-phenylalanine chloromethyl ketone) and TLCK (N-α-p-tosyl-L-lysine chloromethyl ketone) are alkylation reagents that chemically modify the side chain of His or Cys residues in a protein. In search of chemicals that inhibit Nef function, we discovered that TPCK and TLCK alkylated HIV Nef.

**Methods:**

Nef modification by TPCK was demonstrated on reducing SDS-PAGE. The specific cysteine residues modified were determined by site-directed mutagenesis and mass spectrometry (MS). The effect of TPCK modification on Nef-CD4 interaction was studied using fluorescence titration of a synthetic CD4 tail peptide with recombinant Nef-His protein. The conformational change of Nef-His protein upon TPCK-modification was monitored using CD spectrometry

**Results:**

Incubation of Nef-transfected T cells, or recombinant Nef-His protein, with TPCK resulted in mobility shift of Nef on SDS-PAGE. Mutagenesis analysis indicated that the modification occurred at Cys55 and Cys206 in Nef. Mass spectrometry demonstrated that the modification was a covalent attachment (alkylation) of TPCK at Cys55 and Cys206. Cys55 is next to the CD4 binding motif (A_56_W_57_L_58_) in Nef required for Nef-mediated CD4 downregulation and for AIDS development. This implies that the addition of a bulky TPCK molecule to Nef at Cys55 would impair Nef function and reduce HIV pathogenicity. As expected, Cys55 modification reduced the strength of the interaction between Nef-His and CD4 tail peptide by 50%.

**Conclusions:**

Our data suggest that this Cys55-specific alkylation mechanism may be exploited to develop a new class of anti HIV drugs.

## Background

Nef proteins of primate lentiviruses, HIV-1, HIV-2 and SIV, are abundantly expressed in the early phase of HIV-1 infection and play a crucial role in the pathogenicity of HIV-1 and the development of AIDS [1-8]. One prominent piece of evidence is that HIV-1 strains isolated from some long-term survivors carried deletions or truncations of *nef *exclusively [9,10]. The pathological roles of Nef in the development of AIDS have been attributed to several Nef biological activities, including downregulation of the viral primary receptor CD4 [11] and downregulation of the cell surface expression of class-I major histocompatibility complex (MHC-I) [12,13]. Nef also affects T cell activation and apoptosis in favor the viral replication by engaging several signaling molecules, such as Vav, Pak2, ASK1 and Src family kinases [14-18] (for reviews, see [19,20]). Nef has no known catalytic activity; it acts essentially as a connector to link CD4, MHC-I, and possibly some other target molecules to adaptor protein (AP) complexes AP-1, AP-2 or AP-3, responsible for the endocytosis and subsequent lysosomal degradation of Nef's targets. We found that Nef-mediated CD4 downregulation is AP-2 dependent and required an ubiquitinated lysine residue K144 in HIV-1 Nef [21,22]. The structure of HIV-1 Nef has been established by NMR and X-ray crystallography [23-25] (see [26] for a review). HIV-1 Nef protein consists of a conserved core domain of about 120 residues and two flexible regions - the N-terminus 68 amino acids flexible arm and a 32 amino acid loop structure (V148-L181) located in the C-terminal region. The HIV protease cleavage site C_55_AW_57_LEA [27] and CD4 binding motif (A_56_W_57_L_58_) [28] are located in Nef N-terminal region. Nef is myristoylated at a Gly residue (G2) in the N-terminus, which mediates the membrane association of Nef [29]. The core domain is a α-β globular structure responsible for Nef binding to SH3 domain-containing proteins [16,30,31]. The loop in the C-terminal region contains the dileucine motif ExxxLL_160_, which interacts with adaptor protein complexes AP-1, 2, 3 [32-34].

TPCK (N-α-p-tosyl-L-phenylalanine chloromethyl ketone) and TLCK (N-α-p-tosyl-L-lysine chloromethyl ketone) are alkylation reagents that can chemically modify side chains of specific His or Cys residues in some proteins. It is known that TPCK modifies His in the reactive center of serine protease chymotrypsin and trypsin, resulting in enzymatic inhibition (EC_50 _of 20 μM and 80 μM, respectively) [35,36]. TPCK and TLCK also alkylate the sulfhydryl group of the Cys residue in several other proteins, including protein kinase C [37,38], cAMP-dependent kinase [39,40], HPV-18 E7 [41] and human ETS 1 oncoprotein [42]. Alkylation of Cys side chains makes HPV-18 E7 [41] and human ETS 1 oncoprotein [42] migrate faster on SDS-PAGE.

## Methods

### Cells, antibodies and chemicals

SV40 T antigen-transfected human leukemic Jurkat T cells (JTAg) were cultured in RPMI medium supplemented with 10% FCS. For transient expression, plasmid DNA was transfected into the cells using Lipofectamine 2000™(Invitrogen). Anti-HIV-1 Nef rabbit serum was obtained from NIH AIDS Research and Reference Reagent Program. N-tosyl-L-phenylalanine chloromethyl ketone (TPCK), NA-p-tosyl-L-lysine chloromethyl ketone (TLCK) and N-CBZ-Phe-Ala fluoromethyl ketone (Z-FA-FMK) were purchased from Sigma (Saint Louis, MO).

### Plasmids

HIV-1 Nef (NA7)-GFP plasmid kindly provided by Dr. J. Skowronski was subcloned into pcDNA3 to express un-tagged wt Nef (NA7). Nef (G_2_G_3_/AA) mutant was generated by PCR mutagenesis as described before [43]. Nef (NL4-3) was PCR subcloned into pcDNA3 vector with the template of HIV-1 (NL4-3) provirion from NIH AIDS Research and Reference Reagent Program. Nef Cys-to-Ala mutants C55/A, C142/A, C206/A, C55&206/A, C55&142/A, C142&206/A and C55&C142&C206/A (Cys free) were generated by PCR mutagenesis with wt Nef (NA7) plasmid template using Multi-Quick Change Mutagenesis kit (Stratagene). For *E*. *coli *cell expression, wt Nef and Nef mutants were subcloned into pET-30a (+) vector (Novagen) at Nde I/Not I sites. All mutations generated in this study were confirmed by DNA sequencing.

### Analysis of Nef modification in TPCK- or TLCK-treated JTAg cells

Analysis was performed using Nef (NA7) transfected JTAg cells unless otherwise specified. Cells were transfected with Nef plasmid DNA for 16-20 h and treated with TPCK/TLCK (10 μg/ml) for 30 min. Cells (2 × 10^5^) were boiled in 25 μl 2 × SDS sample buffer and loaded to 11% reducing SDS-PAGE. Nef protein was detected by immunoblotting with polyclonal anti-Nef (1:10,000 dilution) at RT for 2 h or at 4°C overnight, followed by ECL anti-rabbit Ab (1:10,000) at RT for 1 h.

### Nef-His protein preparation and in vitro modification

Plasmid encoding Nef-His in pET-30a (+) vector was transformed into *E*. *coli *BL21 cells. The transformed cells were grown in LB medium at 37°C for 16 h, 1: 10 diluted with fresh LB, and induced with IPTG (1 mM) for 3 hours. Four hundred ml of cells were pelleted, washed with PBS and lysed by sonication. Nef-His protein was isolated with a HisTrap column (Amersham Biosciences) or using Ni-NTA agarose beads (QIAGEN). The beads were washed three times in 20 mM Imidazole/PBS. Nef-His was eluted with 250 mM Imidazole, adjusted with PBS to the concentration of UV absorbance (A_280_) = 1.0, and kept at -20°C before use. For *in vitro *modification, freshly prepared Nef-His was incubated with TPCK (10 μg/ml) at RT for 30 min. Twenty μl of samples was resolved by SDS-PAGE. The gels were stained with Coomassie Blue or immunoblotted with anti-Nef.

### Mass spectrometry

Nef-His protein was *in vitro *modified with TPCK as described above. The completion of the modification was confirmed by SDS-PAGE. Fifty μg of the un-modified and TPCK-modified Nef-His proteins were analyzed by MS to determine the molecular weight. For trypsin-digestion, 20 μg of Nef-His was denatured in 0.1 M ammonium bicarbonate at 55°C for 30 min and then digested at 37°C with trypsin at 1:100 (w/w). The samples were subjected to mass spectrometry (MALDI-ToF) at the NYU medical school service center using MS spectrometer Micromass (Waters).

### Fluorescence titration of CD4 tail peptide with HIV-1 Nef

Fluorescein-labeled CD4 tail peptide (Fluorescein-QAERMSQIKRLLSEKKT, residue 403-419) was synthesized by Sigma. Fluorescence emission was recorded with a FluoroMax-2 fluorescence spectrometer (excitation at 492 nm; emission at 516 nm). CD4 tail peptide of 1.0 μM in PBS was analyzed in a stirred cuvette at 25°C. Data were collected after 30 min incubation with Nef-His. Controls incubated with PBS did not show reduction in fluorescence. Experimental signals were expressed as the percentage of fluorescence reduction averaged from three independent measurements. The signals were plotted against total Nef concentration.

### CD spectrum of Nef-His

Nef-His protein of 100 μM (~2.5 mg/ml) in PBS, pH 7.4, was subjected to CD spectrometry analysis. Far-UV CD measurement at 20°C was made on an Aviv 202 CD spectrometer (Lakewood, NJ) in the department of chemistry of NYU. Data were the average of 4-6 accumulations, using scanning wavelength of 260-195 nm, speed of 20 nm/min, bandwidth of 1 nm, and response time of 0.5 s. Data were plotted using the SigmaPlot software.

## Results

### TPCK and TLCK modified HIV-1 Nef expressed in culture T cells

**Figure 1:**
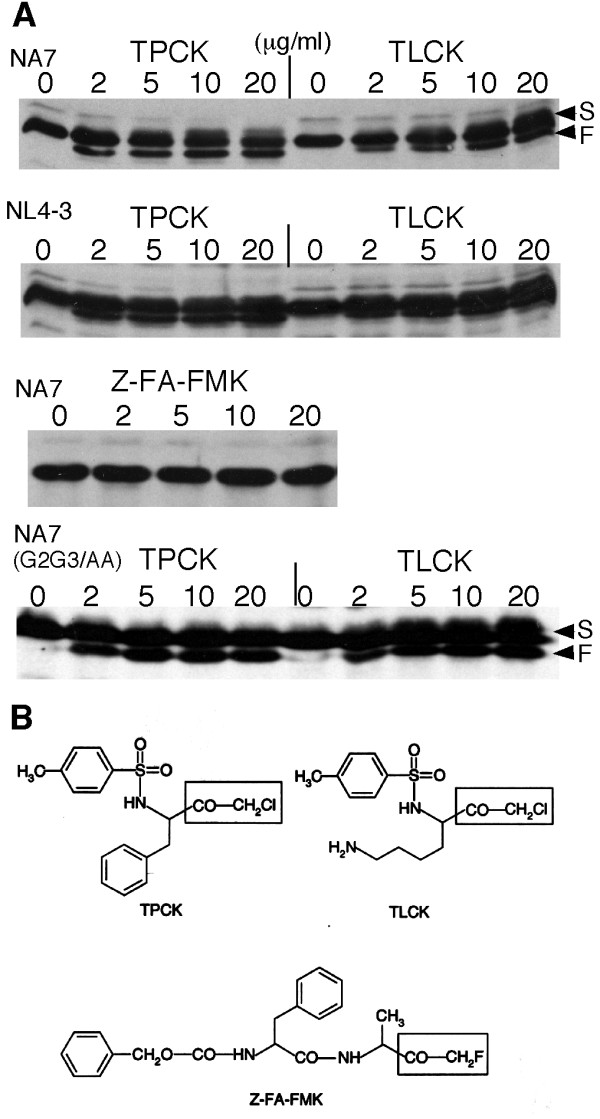
**Treatment of Nef transfected T cells with TPCK or TLCK altered the mobility of Nef on SDS-PAGE**. **(A) **Anti-Nef immunoblotting of Nef proteins from TPCK, TLCK or Z-FA-FMK treated cells. JTAg cells were transfected with Nef NA7 (upper panel), Nef NL4-3 (middle panel) or NA7 (G_2_G_3_/AA), treated with TPCK, TLCK or Z-FA-FMK for 30 min as indicated. The whole cell lysates were immunoblotted with anti-Nef. Arrows indicate the faster (F) or slower (S) migrated Nef proteins. **(B) **Structures of TPCK, TLCK and Z-FA-FMK. The boxed atoms are the alkylating groups reacting with specific His or Cys residues in substrate proteins.

JTAg cells were transfected with plasmids encoding HIV-1 Nef NA7 or NL4-3 and treated with one of the three alkylating reagents, TPCK, TLCK, or Z-FA-FMK. Fig.1A shows that TPCK- or TLCK-treatment altered the mobility of both Nef NA7 and Nef NL4-3 proteins on SDS-PAGE. About 20-30% Nef proteins migrated faster with the treatments (indicated by the letter "F") whereas a small fraction of Nef protein migrated slower (indicated by the letter "S"), which was more noticeable with TLCK than with TPCK. In contrast, treatment with similar doses of Z-FA-FMK did not affect the mobility of Nef protein on SDS-PAGE (Fig.1A). TPCK and TLCK contain chloromethyl ketone whereas Z-FA-FMK contains fluoromethyl ketone (boxed in Fig.1B). The results suggest that Nef proteins may be specifically modified by TPCK and TLCK. TPCK/TLCK at a dose of 1-2 μg/ml (~5-10 μM) was effective in the modification. This dose is lower than the EC50 of TPCK (20 μM) and TLCK (80 μM) in their serine protease inhibition (sigma product information), suggesting a higher reaction specificity of TPCK/TLCK with Nef than with serine proteases. The modification is independent of Nef myristoylation and membrane association since the myristoylation-defective Nef (G_2_G_3_/AA) mutant was also modified with TPCK (Fig.1A bottom).

### TPCK modified Nef at Cys55 and Cys206

**Figure 2:**
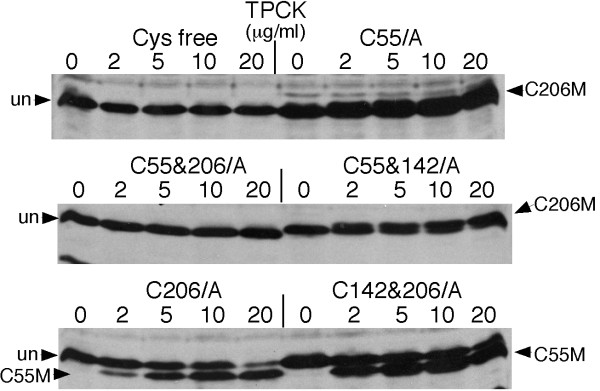
**TPCK-modification of Nef mutants with Cys55, Cys142, and/or Cys206 substituted with Ala**. Plasmid encoding Nef mutant C55/A, C206/A, C142&206/A, C55&206/A, C55&142/A or C55&C142&c206/A (Cys free) were transfected into JTAg cells. TPCK modification was determined as described in Fig. 1. Arrows indicate the Cys206-modified (C206M), Cys55-modified (C55M) and the un-modified Nef (un) proteins.

It was reported that TPCK-treatment altered the mobility of HPV-18 E7 and human ETS 1 oncoprotein on SDS-PAGE as a result of Cys alkylation [41,42]. HIV-1 Nef contains two conserved Cys residues (Cys55 and Cys142) and a partially conserved C-terminal Cys (Cys206) [44]. To find out whether Nef was also modified at Cys residues, we examined the mobility of TPCK-treated Nef Cys mutants on SDS-PAGE. Fig. 2 shows that TPCK-treatment did not cause any mobility shift of Cys-free Nef mutant (upper left panel), suggesting that Cys residues were the residues to be modified. Double Cys mutant C55&206/A showed no mobility shift either (middle left panel), suggesting that Cys55 &/or Cys206, but not Cys142, were the residues modified. Further more, single cysteine mutant C55/A migrated slower on SDS-PAGE, indicating that the modification at Cys206 resulted in a slow migration form of Nef (C206M) whereas Nef mutant C206/A migrated faster on SDS-PAGE (bottom panels), indicating that the modification at Cys55 resulted in a faster migration form (C55M). The migration patterns of Nef mutant C142&206/A (Cys55 modified) and C55&142/A (Cys206 modified) were the same as that of C206/A and C55/A. Taken together, the mutagenesis data suggest that Cys55 and Cys 206 but not C142 and His residues are modified by TPCK. This conclusion is directly proved by the following MS analysis.

### TPCK modified recombinant Nef-His protein in vitro and the modification appeared to be dependent on Nef conformation

**Figure 3:**
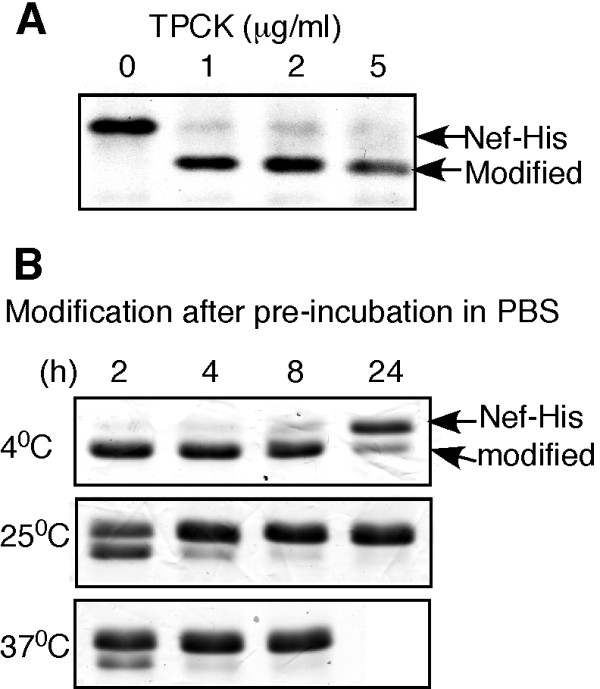
***In vitro *modification of Nef-His protein by TPCK **E. *coli-*expressed Nef-His protein was isolated using Ni-beads as described in Methods. Twenty μl of freshly prepared Nef-His protein at the concentration of ~0.5 μg/μl was incubated with TPCK in PBS at RT for 30 min and then resolved by SDS-PAGE. The gels were stained with Coomassie Blue. **(A) **TPCK-modification of the freshly prepared Nef-His protein at different TPCK concentrations. (**B**) TPCK-modification of the Nef-His protein pre-incubated in PBS at different temperatures for different length of times.

Next we asked whether TPCK-modification of Nef is a direct chemical reaction. The E. *coli *expressed, isolated Nef-His protein was incubated with TPCK in PBS. Fig. 3A shows that Nef-His protein was modified with TPCK *in vitro*, resulting in a faster mobility shift on SDS-PAGE. The results indicated that the modification is a direct chemical reaction between Nef and TPCK. Notably, we found that the freshly prepared Nef-His protein was modified efficiently, with a yield of ~80 to 95%. But the modification yield was greatly decreased if Nef-His protein in PBS had been kept at 4°C for 1-2 days before incubation with TPCK. At higher temperature of 25°C or 37°C, an 8 or 4 h pre-incubation was enough to almost abrogate the modification (Fig. 3B). As shown in the figure, Nef-His was not degraded during the incubation. Since it is known that the isolated recombinant Nef protein is unstable and undergo conformational change &/or aggregation at higher concentrations [24], the results suggested that a potential conformational change of Nef may affect its modification with TPCK. It is possible that the overexpressed Nef protein in culture cells also undergoes conformational change &/or aggregation, which could explain why Nef was only partial modified with TPCK (Fig.1). Supporting this notion, we observed that alkylation efficiency of Nef in culture cells was reduced when the new Nef protein synthesis was stopped by addition of cycloheximide and MG132 in cell culture for several hours (data not shown).

### MS analysis proved that TPCK was covalently bound to Cys 55 and Cys 206 but not to His residues

**Figure 4:**
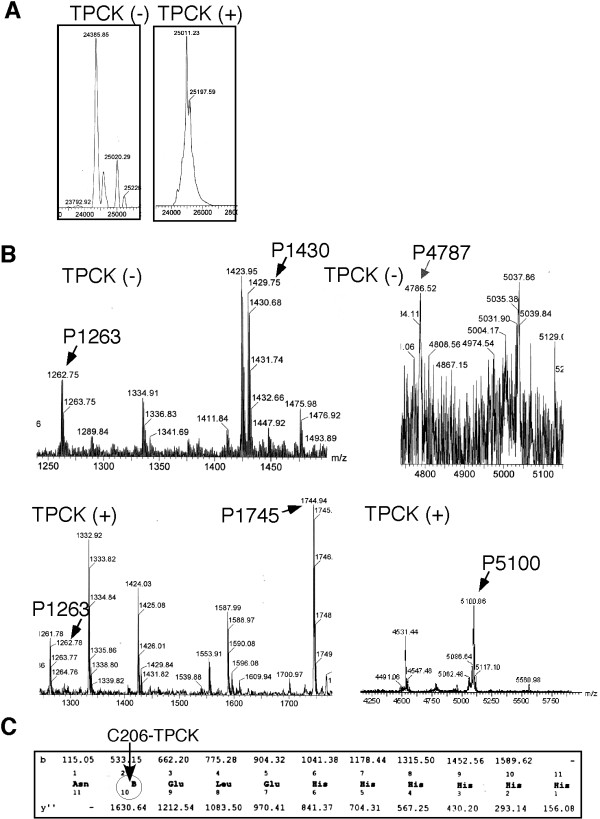
**Mass Spectrometry (MS) of the unmodified and TPCK-modified Nef-His proteins**. **(A) **MS- determination of the molecular weight of the unmodified (TPCK-) and modified (TPCK+) Nef-His proteins. **(B) **Tryptic mapping of Nef-His proteins by MS. Unmodified (top panel) or TPCK-modified Nef-His proteins (bottom panel) were excised from SDS PAGE gels, digested by trypsin and injected into Micromass (Waters) for MS (MALDI-ToF). Arrows indicate the tryptic peptides containing cysteine: P1263 (C142), P1430 (C206) and P4787 (C55) from unmodified Nef-His (top panel), and P1263 (C142), P1745 (C206) and P5100 (C55) from the TPCK-modified Nef-His (bottom panel). **(C) **MS sequencing of the modified C-terminal peptide (P1745). Residue 10^B ^is the modified Cys206. Three residues Glu, Leu and Glu between Nef and His-tag are translated from the vector poly-linker region. Note, different sensitivity scales are used to show the unmodified C55 (P4787) and TPCK modified C55 (P5100).

To prove that TPCK is covalently bound to Cys55 and Cys206 and to rule out that TPCK may modify some other Nef residues unaffecting Nef's mobility, we analyzed the TPCK-modification of Nef-His by mass spectrometry (MS). Fig. 4A shows the molecular weight of untreated and TPCK-treated Nef-His determined by MS. The peak of untreated Nef-His is 24386 Dalton (TPCK-) whereas TPCK-treated Nef-His is 25011 Dalton (TPCK+). The 631 Dalton difference equals the molecular weight of two TPCK molecules (2 × 352 Dalton) minus two HCl molecules (2 × 36.5 Dalton), indicating that each Nef molecule was covalently bound with two TPCK molecules. To prove that TPCK was bound to Cys55 and Cys206, we did a tryptic mapping (Fig. 4B). Amino acid sequence of Nef-His predicts that tryptic peptide of 1430 Da (P1430) contains Cys206, tryptic peptide of 4787 Da (P4787) contains Cys55, and tryptic peptide of 1263 Da (P1263) contains Cys142. All these peptides were detected (indicated by arrows) in untreated Nef-His. With TPCK-modification, P1430 and P4787 were converted to P1745 and P5100. TPCK-treatment did not affect P1263, indicating that Cys142 is not alkylated. Note, we have to use a high sensitivity scale for detection of P4787 (up right panel) due to its low UV absorbance. With the attachment of TPCK (N-α-p-tosyl-L-phenylalanine chloromethyl ketone) - a highly UV detectable chemical, P5100 (4787+TPCK) and P1745 (1430+TPCK) (bottom panels) exhibited a much higher UV absorbance. We also sequenced the tryptic peptide P1715 that contains the very C-terminal His-tag and Cys206 (Fig. 4C). The results showed that none of the His residues in His-tag was alkylated, whereas Cys206 was. Residue B of the peptide (Cys206, circled in Fig. 4C) had a molecular weight of 418 Da, exactly equal to that of a one TPCK-alkylated Cys. Thus, the collective MS data proved that TPCK alkylates Cys55 and Cys206 but not Cys142 or any His residues.

### TPCK alkylation at Cys55 severely impaired Nef's interaction with CD4 tail peptide

**Figure 5:**
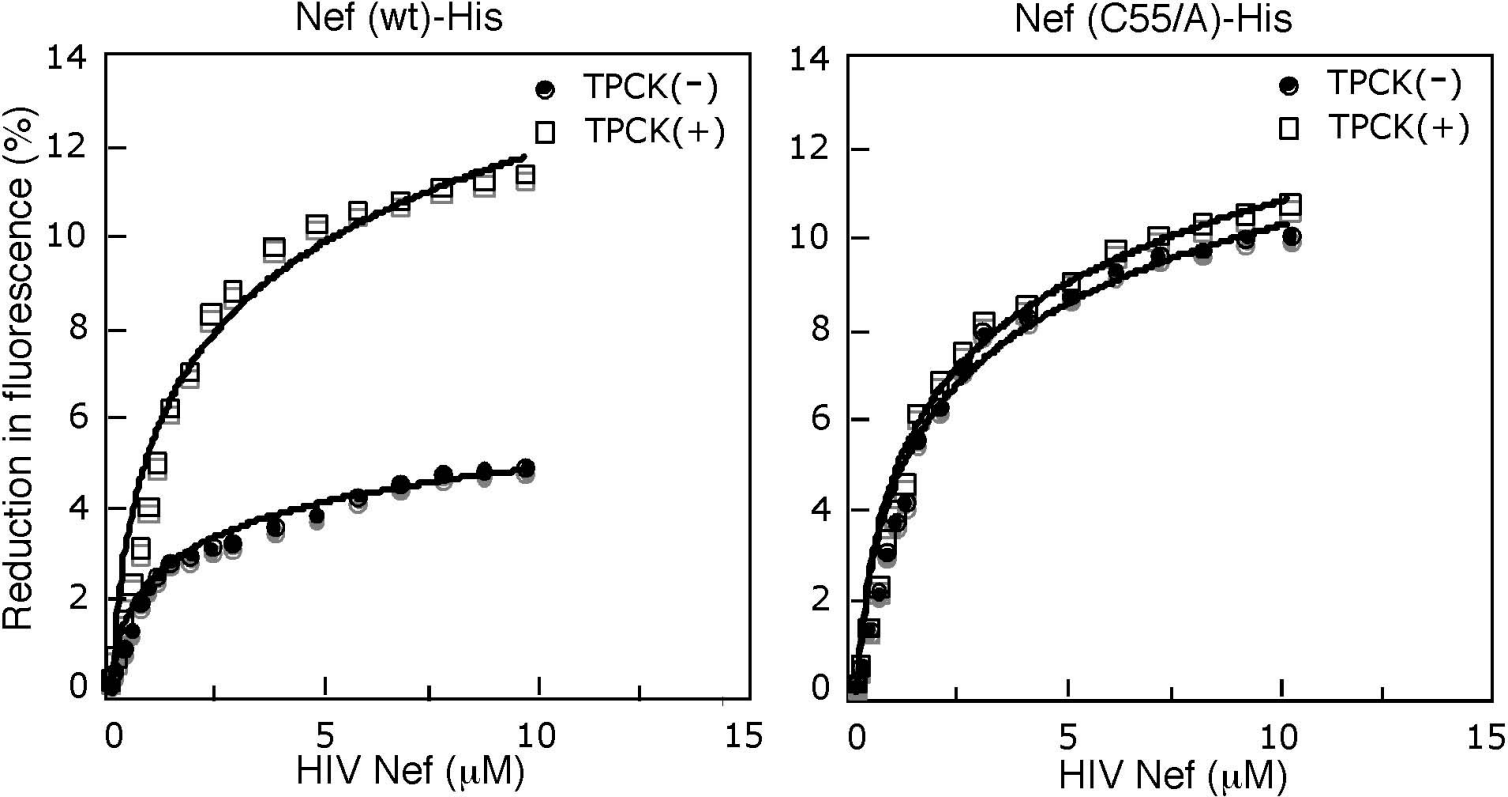
**Titration of a fluorescein labeled CD4 tail peptide with HIV Nef-His proteins**. 1.0 μM of the CD4 peptide in PBS was incubated for 30 min with Nef-His proteins at the concentrations from 0.01 to 10 μM in 0.5 ml volume. Fluorescence emission was recorded with a FluoroMax-2 fluorescence spectrometer (excitation at 492 nm; emission at 516 nm) in a stirred cuvatte at 25°C. Reduction in fluorescence emission after incubation with a protein is expressed as the percentage of the fluorescence before incubation. The reduction in fluorescence is plotted against Nef-His concentration. The values are the average of three repeats. Left panel: Fluorescence reduction of the CD4 peptide after incubation with unalkylated Nef (wt)-His (black circle) or TPCK-alkylated Nef (wt)-His (white square). Right panel: Fluorescence reduction of the CD4 peptide incubated with the untreated Nef (C55A)-His (black circle) or TPCK-treated Nef (C55A)-His (white square).

Cys55 is next to Nef motif A56W57L58, a site implicated in the interaction of Nef with CD4, Nef-mediated CD4 downregulation and the onset of AIDS [9,10,28]. To ask whether the attachment of a bulky TPCK molecule to Cys55 affects Nef-CD4 interaction, we performed an *in vitro *CD4-Nef binding assay following a published protocol [45]. In the assay, a fluorescein-labeled 17 amino acid CD4 tail peptide was incubated with Nef-His or TPCK modified Nef-His proteins at increase concentrations. Quenching of the fluorescence emission from the label CD4 peptide by Nef-His proteins was measured as the results of CD4-Nef interaction [45]. Fig. 5 (left panel) compares the titration curve of the unmodified wt Nef-His with that of TPCK-modified Nef-His. The results showed that the fluorescence emission was quenched by 11.6% with unmodified Nef-His protein (10 μM) whereas was quenched by 4.9% with TPCK-modified Nef-His, indicating that TPCK-modification resulted in more than 50% of decrease in the strength of Nef-CD4 interaction. To confirm that the effects are C55 modification specific, we also compared the titration curve of the unmodified Nef mutant (C55/A)-His with that of TPCK-treated Nef (C55/A)-His. Fig. 5 (right panel) shows that the titration curves of the untreated and treated (C55/A)-His were quite similar. At 10 μM concentration, the level of quench was 10.6% and 9.8% for untreated and treated, respectively, confirming that the effects are depended on modification of Nef C55. We concluded that the alkylation at Cys55 will greatly impair Nef-CD4 interaction and, therefore, would weaken HIV-1's pathogenicity. In addition, the fluorescence reduction by wt Nef-His was 11.6% compared with 10.6% by Nef (C55/A)-His, suggesting that C55A mutation itself may have a weak effects on Nef-CD4 interaction.

### CD spectrometry data indicated a moderate Nef conformational change after TPCK alkylation

**Figure 6:**
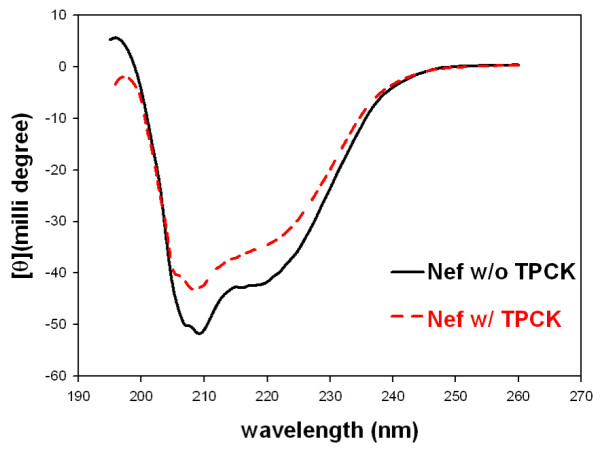
**CD spectra of untreated Nef-His or TPCK alkylated Nef-His**. The experiment was described in Methods. Samples were scanned at 260-195 nm (far-UV) at 20°C on an Aviv 202 CD spectrometer (Lakewood, NJ). Acquired data were plotted using the SigmaPlot software.

To ask whether alkylation alters the solution structure of Nef, we compared the CD spectrometry of Nef-His proteins unmodified or modified with TPCK (Fig. 6). The CD spectrometry showed that Nef has an overall α-β structure with an absorbance of α-helix at 208 nm and absorbance of β-sheet at 216-220 nm. TPCK alkylation did not result in a shift of the absorbance wavelength (nm), suggesting that there was no global change in the overall α-β structure. However, the α-helix absorbance at ~208 nm apparently became weaker, suggesting a less stable Nef structure upon alkylation. This is consistent with our observation that TPCK-treatment reduced Nef half-life in cultured T cells (not shown). Therefore, in addition to attenuation of Nef-CD4 interaction, this may be as a second mechanism for alkylation to undermine Nef's function.

## Discussion

This study demonstrated that alkylation reagents, TPCK and TLCK, modify HIV-1 accessory protein Nef in live T cells and *in vitro*. Mutagenesis and MS analysis indicated that TPCK-modification of Nef is an alkylation reaction that resulted in the covalent bound of TPCK molecule to the side chains of Cys55 and Cys206 residues (Fig. 1, 2, 3, 4). Several lines of evidence suggest that the reaction is quite specific: (1) TPCK and TLCK have been used as specific serine protease inhibitors. The EC_50 _values of TPCK and TLCK alkylation on Nef are lower than that on chymotrypsin and trypsin, suggesting higher alkylation specificity than that of serine proteases. (2) Z-FA-FMK, a structurally very similar alkylation reagent, is inactive in modifying Nef (Fig. 1). (3) TPCK reacts with Cys but not with His residues, including those in the C-terminal His-tag, fully accessible to TPCK (Fig. 4). (4) TPCK appears to alkylate Cys55 more efficiently than to Cys206 (Fig.1).

The mechanism by which TPCK alkylates Cys residue is much less understood than the mechanism by which it alkylates His residues. It is well known that TPCK inhibits serine proteases by alkylating the His side chain at an enzyme' reactive center [35,36]. This understanding has rationalized the use of TPCK in signal transduction studies. In addition, some recent reports implicated the effects of alkylation at Cys, rather than at His residues [46-48]. However, how TPCK reacts with specific His or Cys is unclear. Our study showed that in case of Nef, the accessibility of Cys residues for TPCK appeared important but not sufficient for TPCK-modification. The TPCK-modified Cys55 and Cys206 are both accessible, locating in Nef N-terminal flexible region and at the C-terminal end, respectively, whereas the none-modified C142 is buried in the Nef core [26]. However, accessibility cannot explain why TPCK did not react with any His residues despite that there are nine His residues in Nef, of which several are accessible. They include His 40 in the N-terminal flexible region and His166/His171 in the C-terminal loop region. In addition, TPCK did not react with any His residues in the C-terminal His-tag. Probably the residues surrounding the reactive Cys or His are involved in the interaction with TPCK side chain, thus contributed to alkylation specificity.

Cys55 is next to Nef motif A56W57L58, a site important for Nef-CD4 interaction and development of AIDS [28]. The motif is also the cleavage site for HIV protease [27]. It is conceivable that the covalent attachment of a bulky TPCK molecule to Cys55 would interfere with Nef-CD4 interaction and some other Nef functions. Fluorescence titration data indicated that TPCK-modification indeed dramatically reduced the binding strength of Nef to a CD4 tail peptide (Fig. 5). TPCK-modification may have an additional mechanism against HIV-1 by altering Nef conformation as shown by the CD spectrum change (Fig. 6) and making it unstable as suggested by a shortened half-life of Nef in T cells also (unpublished data). Unfortunately, current cell system is not fit for testing anti HIV-1 activity due to technical difficulty. TPCK only partially (50%, maximum) alkylates wt Nef overexpressed in cultured T cells, leaving more than half of Nef without alkylation (Fig.1). A small fraction of unalkylated Nef protein is sufficient to downregulate CD4. Moreover, TPCK is toxic to T cells at high concentrations, which compromises the interpretation of an anti HIV-1 activity.

Our finding suggests that TPCK can serve as a prototype of a class of drugs that retains the Cys55 modification activity but has desired pharmacodynamic and pharmacological properties. A 3-D structure of the TPCK-bound Nef could guide the design and synthesis of new compounds. In this regard, we have developed a convenient method of generating large quantity of TPCK-bound Nef for structure studies (Fig. 3, 4). A comparison of such a 3-D structure with the existing 3-D model of TPCK bound to a His residue at the catalytic center of a serine protease [49] may aid the development of similar compounds that are specific for cysteine over histidine or vice versa.

## Conclusions

Chloromethyl ketone reagents TPCK and TLCK directly react with Cys55 and Cys206 in Nef. TPCK alkylation at Cys55 dramatically weakens Nef-CD4 interaction, suggesting that TPCK-like small chemicals with better pharmacokinetics and pharmacodynamics may be developed for HIV disease intervention.

## List of abbreviations

HIV: human immunodeficiency virus; JTAG: SV40 large T antigen-transfected human leukemic Jurkat T cells; TPCK: N-α-p-tosyl-L-phenylalanine chloromethyl ketone; TLCK: N-α-p-tosyl-L-lysine chloromethyl ketone; Z-FA-FMK: N-CBZ-Phe-Ala fluoromethyl ketone; MHC-I: major histocompatibility complex class I.

## Competing interests

The authors declare that they have no competing interests.

## Authors' contributions

YJ is the principal investigator in this study. XZ participated in its design and helped to draft the manuscript. YC carried out the CD spectrometry study. SJB involved in data analysis and revision of the manuscript. All authors read and approved the final manuscript.
